# Uptake dynamics in the Lactose permease (LacY) membrane protein transporter

**DOI:** 10.1038/s41598-018-32624-7

**Published:** 2018-09-25

**Authors:** D. Kimanius, E. Lindahl, M. Andersson

**Affiliations:** 1 Department of Biochemistry and Biophysics, Science For Life Laboratory, Stockholm University, SE-106 91 Stockholm, Sweden; 2 Department of Chemistry, Umeå University, SE-901 87 Umeå, Sweden

## Abstract

The sugar transporter Lactose permease (LacY) of *Escherichia coli* has become a prototype to understand the underlying molecular details of membrane transport. Crystal structures have trapped the protein in sugar-bound states facing the periplasm, but with narrow openings unable to accommodate sugar. Therefore, the molecular details of sugar uptake remain elusive. In this work, we have used extended simulations and metadynamics sampling to explore a putative sugar-uptake pathway and associated free energy landscape. We found an entrance at helix-pair 2 and 11, which involved lipid head groups and residues Gln 241 and Gln 359. Furthermore, the protein displayed high flexibility on the periplasmic side of Phe 27, which is located at the narrowest section of the pathway. Interactions to Phe 27 enabled passage into the binding site, which was associated with a 24 ± 4 kJ/mol binding free energy in excellent agreement with an independent binding free energy calculation and experimental data. Two free energy minima corresponding to the two possible binding poses of the lactose analog β-D-galactopyranosyl-1-thio-β-D-galactopyranoside (TDG) were aligned with the crystal structure-binding pocket. This work outlines the chemical environment of a putative periplasmic sugar pathway and paves way for understanding substrate affinity and specificity in LacY.

## Introduction

Transport across cellular lipid bilayers is central to a wide range of physiological processes, such as nutrient uptake, waste removal, and upholding of gradients. Membrane proteins interspersed in the lipid bilayer undergo transitions between inward-open and outward-open conformational states, which affect the free energy landscape associated with substrate binding and unbinding and ultimately results in transport of the substrate. X-ray crystallography has produced several high-resolution structures of transporters trapped both in inward-open and outward-open states^1^. A key question is how protein conformational change is coupled to substrate binding and release. Monitoring the structural pathways explored by the substrate upon entering and exiting the protein can putatively add to our understanding of substrate affinity and specificity. However, the transient nature of the protein-substrate interactions displayed in substrate binding or release and involvement of lipids frequently aggravates experimental characterization. Because molecular dynamics (MD) simulations can extract transient structural and energetic features of proteins in complex lipid environments associated with substrate transport, such computational methods offer an attractive alternative to study membrane transport.

**Figure 1:**
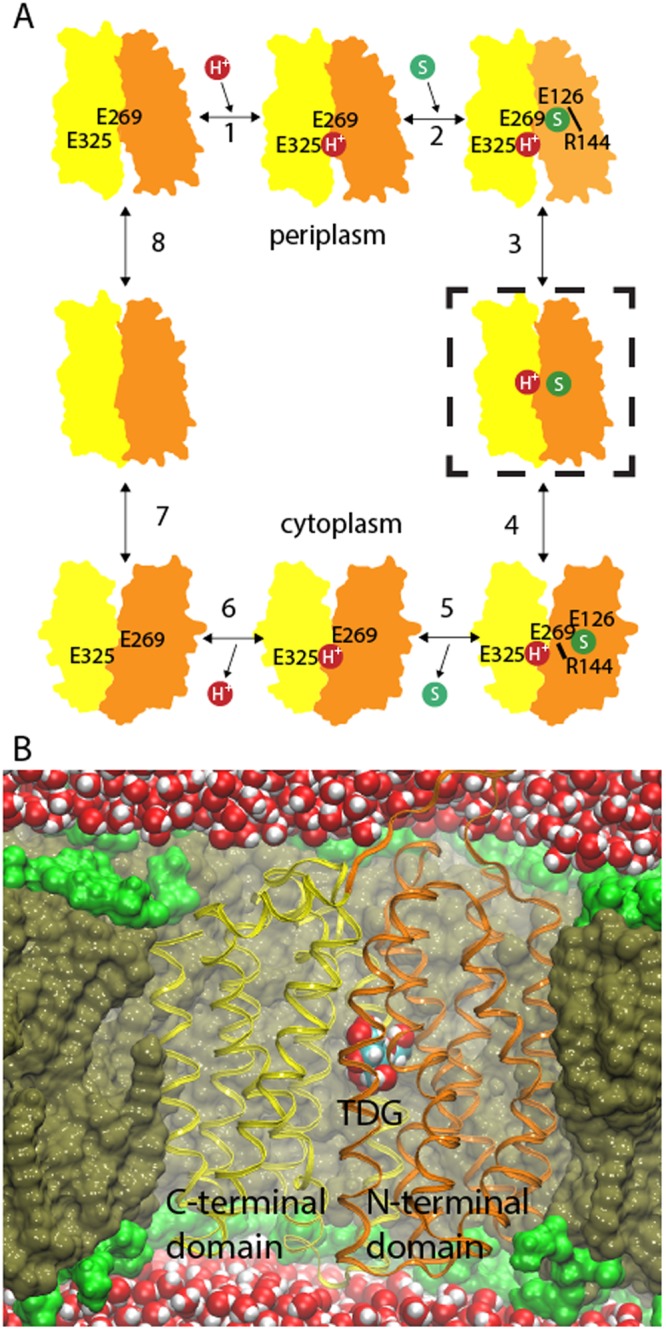
LacY structure and mechanism (**A**) The general reaction scheme of LacY galactoside/H^+^ symport, where a proton (step 1) and a substrate molecule (step 2) bind to a LacY state open towards the periplasm, which changes accessibility to the cytoplasm (step 3–4). Substrate release (step 5) and deprotonation (step 6) trigger reformation of the periplasmic open state (step 7–8). The simulated state is marked with a dashed box. (**B**) The TDG-bound LacY crystal structure (PDB ID: 4OAA) inserted into a POPE lipid bilayer and solvated by water.

The Lactose permease of *Escherichia coli* (LacY) has evolved into a prototype system for understanding membrane transport. LacY catalyzes galactopyranoside transport using a proton gradient across the cytoplasmic membrane^2,3^ (Fig. 1A). The LacY structure was first captured in an inward-facing state that was closely sealed towards the periplasm^4–7^. Because LacY states open to the periplasm have eluded structural determination, outward-facing models were suggested based on computer simulations^8,9^ or by swapping conformations of inverted-topology repeats^10^. Finally, an engineering approach that replaced two periplasmic Gly residues with Trp^11^ enabled high-resolution structures of a state closed towards the cytoplasm with a narrow periplasmic pathway in the presence of the bound high-affinity lactose analogs 4-nitrophenyl-α-D-galactopranoside (NPG)^12^ or β-D-galactopyranosyl-1-thio-β-D-galactopyranoside (TDG)^13^. Recently, the structures of states stabilized by single-domain camelid nanobodies in outward-open conformations were presented^14,15^. In contrast to beliefs that opening and closing the transporter must necessarily involve large structural change, relatively small rearrangements accompanied the periplasmic opening. A region in helix 1 displayed a kink compared to the sugar-bound structures, which resulted in a constriction region lined by Phe 27, whose role is not clear since the F27C mutant shows normal transport activity^16,17^. In addition, a central binding site has been defined^18^ that was almost identical in all periplasmic-facing structures^12–14^. Hence, while we now have access to detailed protein structural information, it is not clear how sugar molecules reach the internal binding site from the periplasm. Determination of the chemical environment experienced by the transported sugar might give clues to substrate affinity and specificity. In addition, in accordance with a sugar/H^+^ symport mechanism, there is a strict requirement for the LacY protein to be protonated in order to bind the sugar substrate. Proton binding in the sugar-bound state has been attributed to Glu 325^3^. Therefore, to characterize protein-sugar dynamics in a solvated periplasmic pathway, we simulated the TDG-bound crystal structure in a 1-palmitoyl-2-oleoyl-*sn*-glycero-3-phosphoethanolamine (POPE) lipid bilayer with Glu 325 protonated (Fig. 1B).

The LacY transporter is a particularly attractive target for studying substrate interactions due to the availability of high-resolution crystal structures^4–7,12–14^ and a wealth of biochemical data^2,3^. The protein structural dynamics and sugar interactions in the inward-open states have been thoroughly characterized using computer simulation^19–22^. A first attempt to predict the periplasmic pathway pulled artificially a lactose molecule through a LacY structure, which was sealed towards the periplasm^23^. Using less intrusive methods, Pendse *et al*. modelled sugar dynamics in a periplasmic-open state^8^. However, limited sampling obscured determination of a putative periplasmic pathway. Now, with high-resolution structural information of sugar-bound outward-facing states at hand and specialized simulation hardware, it is possible to identify a LacY periplasmic pathway and to determine the molecular basis of sugar uptake.

In this work, we simulated the TDG-bound LacY structure in a POPE lipid bilayer and observed significant protein structural change and spontaneous release of TDG into bulk water followed by re-entering into the internal binding pathway. Increased levels of sugar-lipid interactions accompanied the release/uptake events at the protein-water interface. The simulated TDG coordinates served as starting positions for metadynamics simulations that enabled determining the free energy landscape associated with the putative periplasmic pathway. The crystallographic binding position coincided with two free energy minima, which reflects the two possible binding poses of the TDG disaccharide. Based on residue-interaction frequencies, we suggest the following putative uptake mechanism: residues Gln 241 and Gln 359 guide the substrate between TM helices 2 and 11 towards Phe 27, which attaches to one of the sugar rings and shuttles the substrate into the internal binding site after overcoming a 20 ± 5 kJ/mol free energy barrier where it is locked into place by primarily Glu126. The observed binding free energy of −24 ± 10 kJ/mol was in excellent agreement with independent binding free energy calculations (−24 ± 4 kJ/mol) and experimental data (−23 kJ/mol)^24^.

## Results

### The periplasmic end of LacY is highly dynamic and open in the lipid bilayer

**Figure 2:**
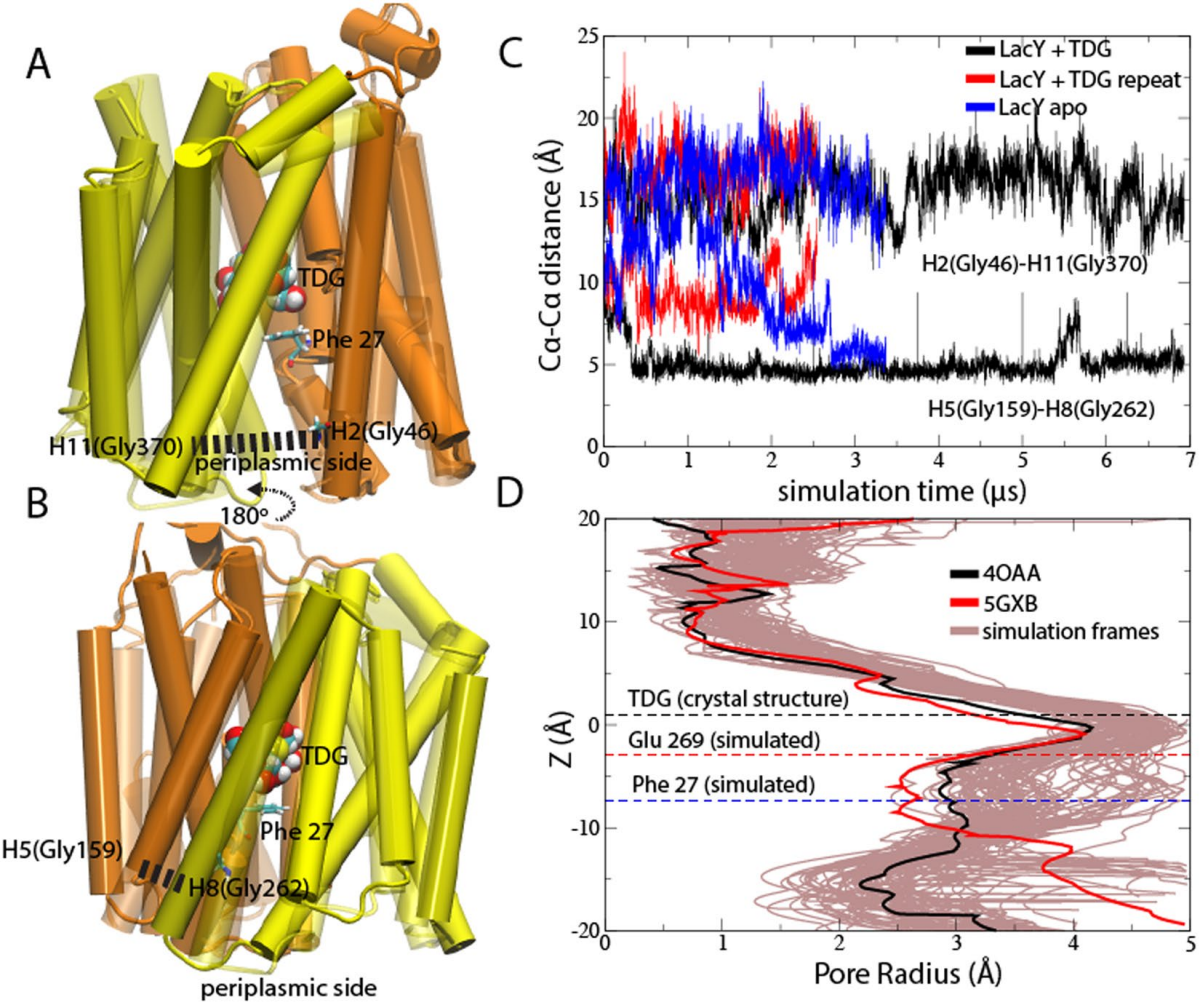
Simulated structural rearrangements in the sugar-bound state. The crystal structure (transparent) and last frame from the simulation (solid) show the helical simulated shifts of (**A**) helices 2 and 11 and (**B**) helices 5 and 8. The TDG molecule and Phe 27 are shown in their crystallographic positions. (**C**) The Cα-Cα distances in helix pair 2 (Gly 46) – 11 (Gly 370) and helix pair 5 (Gly 159) – 8 (Gly 262) for the sugar-bound simulation, the repeat simulation, and the apo state simulation. (**D**) One-hundred pore radii extracted from the final 100 ns of the sugar-bound simulation (gray). The pore radii from the occluded (PDB ID: 4OAA) and periplasmic open (PDB ID: 5GXB) crystal structures are shown in black and red, respectively. Dashed lines correspond to the TDG position in the crystal structure (black) and the average simulated centers-of-mass of residues Glu 269 (red) and Phe 27 (blue).

To explore dynamics at the periplasmic opening and internal substrate-binding site, we mutated the double-Trp structure with bound TDG (PDB ID: 4OAA) back to wild-type, modelled the missing 191–206 cytoplasmic loop, and inserted it into a POPE lipid membrane. The Anton supercomputer with simulation-dedicated hardware^25^ was used to simulate two identical LacY systems with bound TDG with different random seed (6.9 μs and 2.5 μs, respectively) as well as the apo protein (3.5 μs). The overall protein dynamics in all three simulations generated root means square deviation’s (RMSD) of ~3 Å (Supplementary Fig. S1A), which is usually considered stable systems. The root mean square fluctuations (RMSF) also displayed very similar dynamics, except for the loop regions, as can be expected (Supplementary Fig. S1B). Inspection of the Trp-to-Gly mutant positions 46 (helix 2) and 262 (helix 8) showed helix 8 to display elevated dynamics compared to helix 2 in all three simulations. We therefore monitored Cα-Cα distance between helices 2 and 8 and their neighboring helices in the opposite terminal domain (helices 11 and 5, respectively) (Fig. 2A,B). In the TDG and apo simulations, helix 8 made ~7 Å displacements to seal the helix 5–8 interface (Fig. 2C). While the repeat simulation also decreased the distance between helices 5 and 8, a tight seal was not achieved. In agreement with the moderate RMSF at position 46, the helix pair 2 and 11 remained equally open as in the TDG-bound crystal structure in all three simulations.

The pore radii of the TDG-bound and periplasmic-open crystal structures are both tightly sealed towards the cytoplasm and display central binding cavities that are separated from the periplasmic funnel by Phe 27 (Fig. 2D). Furthermore, the periplasmic-open crystal structure is more open towards the periplasm, but only distal to Phe 27. We calculated pore radii from 100 simulation frames extracted from the final 100 ns of the TDG simulation. All protein structures were sealed towards the cytoplasm and showed very similar central binding cavities. In contrast, below Phe 27 the structural dynamics were highly elevated generating pore radii ranging between 2 Å and >5 Å. Clearly, the outer periplasmic parts of LacY – at the level of Phe 27 – are capable of dramatic structural rearrangements.

### Identification of a putative substrate-uptake pathway

**Figure 3:**
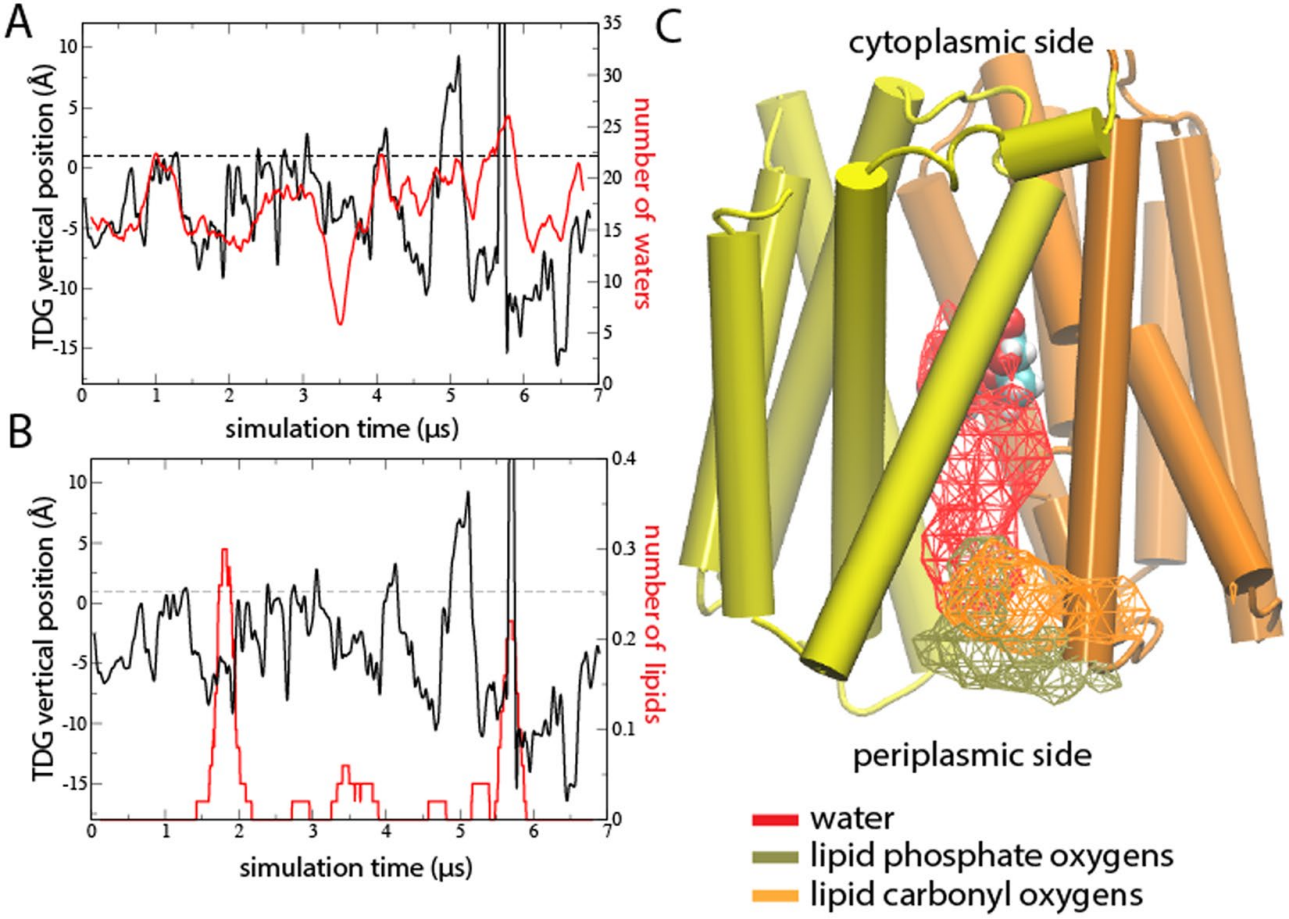
Sugar interactions and transient release and re-uptake. The number of TDG interactions within 3.5 Å to (**A**) water and (**B**) lipids (red) are shown along the corresponding center-of-mass position of the TDG molecule along the membrane vertical (z) (black). Dashed lines correspond to the center-of-mass of TDG in the crystal structure. (**C**) Iso-density surfaces at 1.2% based on TDG interactions within 4 Å to either phosphate oxygens (tan) or carbonyl oxygens (orange) of the lipids and water (red) are shown with respect to the equilibrated LacY structure and TDG.

During the 50 ns equilibration simulation, the center-of-mass vertical position of the TDG molecule was aligned with the internal binding pocket observed in the crystal structure (Supplementary Fig. S2). In this position, the TDG was solvated by on average 18 water molecules, the protein interactions varied in-between zero and five contacts, and no interactions to lipids were observed. In contrast, the extensive sampling in the Anton production enabled monitoring the bound TDG molecule to break free of the internal binding site to further explore the periplasmic cavity (Fig. 3). At ~5.6 μs, the TDG molecule exited the protein and was released into the surrounding bulk water followed by re-entry within 100 ns. During the final 1 μs following re-entry the TDG molecule re-established itself 2.5 Å from the crystallographic binding position. By tracking TDG-water interactions, the sugar was found to be relatively well hydrated also in vicinity of the binding pocket. However, the TDG exit and re-entry was accompanied by the expected increase in water solvation (Fig. 3A). We also observed bursts of lipid interactions accompanying periods where TDG was located close to the periplasmic opening (Fig. 3B). Because lipid interactions were observed also during the exit and re-entry processes and formed part of the observed sugar pathway (Fig. 3C), lipids may assist in sugar uptake. In addition, occasional dips in TDG hydration coincide with periods of lipid interactions, which further illustrates the interplay between lipids, water and the substrate. While the full lipid head group (i.e. phosphate and carbonyl oxygens) contributes to binding TDG, no interactions to the acyl chain lipid tails were observed (Fig. 3C). Importantly, tracking TDG, water, and lipid dynamics during the simulation enabled identification of a putative periplasmic pathway capable of spontaneous substrate release and uptake.

**Figure 4:**
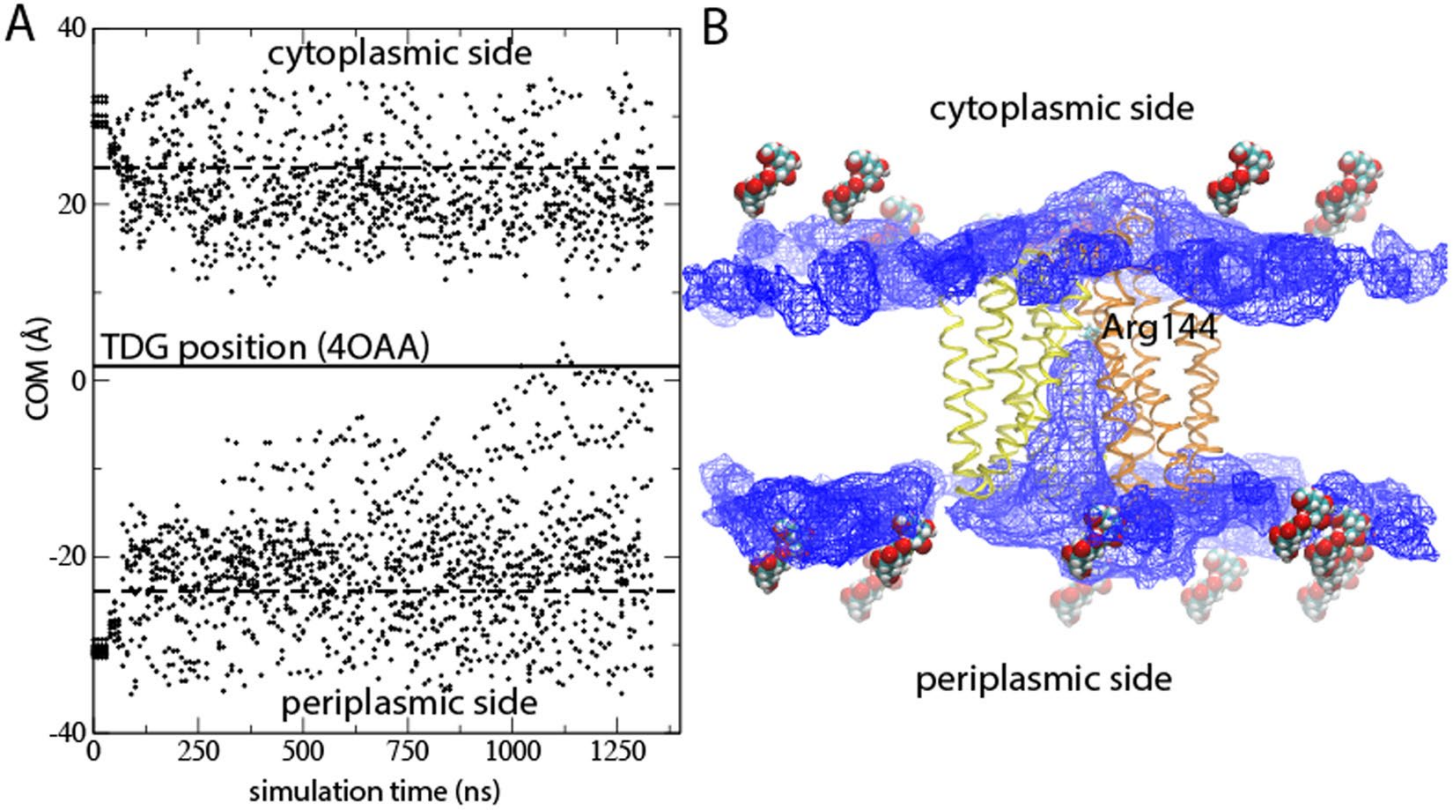
Simulation of sugar uptake from periplasmic bulk (**A**) Simulated center-of-mass (COM) positions of 19 TDG molecules initially positioned randomly in cytoplasmic and periplasmic bulk at least 10 Å from the protein boundaries (dashed lines). The solid line corresponds to the COM position of TDG in the 4OAA crystal structure. (**B**) The simulated TDG positions visualized by an iso-density map at 1.2% mapped onto the final frame of the Anton production simulation. The Arg 144 side chain marks the position of the crystallographic binding site. The initial positions of the 19 TDG molecules are shown in van der Waal spheres.

To verify the identified periplasmic pathway, we placed multiple TDG molecules in bulk water and performed a 1.3 μs control simulation starting from the last frame of the extended TDG simulation. Indeed, after >250 ns simulation time a TDG molecule entered from the periplasm (Fig. 4A). During the remaining microsecond the TDG molecule reached the crystallographic binding site (Fig. 4B). As expected, the tightly sealed protein prevented TDG molecules from entering from the cytoplasm.

### The free energy profile associated with the simulated periplasmic pathway

To obtain sufficient sampling for determining free energies associated with sugar uptake, multiple unbinding and binding events would be required. Therefore, to determine the free energy landscape experienced by the TDG molecule in this simulated state of LacY, we instead turned to metadynamics simulations that can enhance sampling along a collective variable (CV). Here, two complementary collective variables were used based on the RMSD distance to six TDG reference positions (extracted from the sugar-bound Anton simulation) spread along the path between the internal binding site and outside the periplasmic opening^26^. The resulting free energy landscape was based on sampling statistics from six parallel simulations adding up to a total of 1.4 μs simulation time.

**Figure 5:**
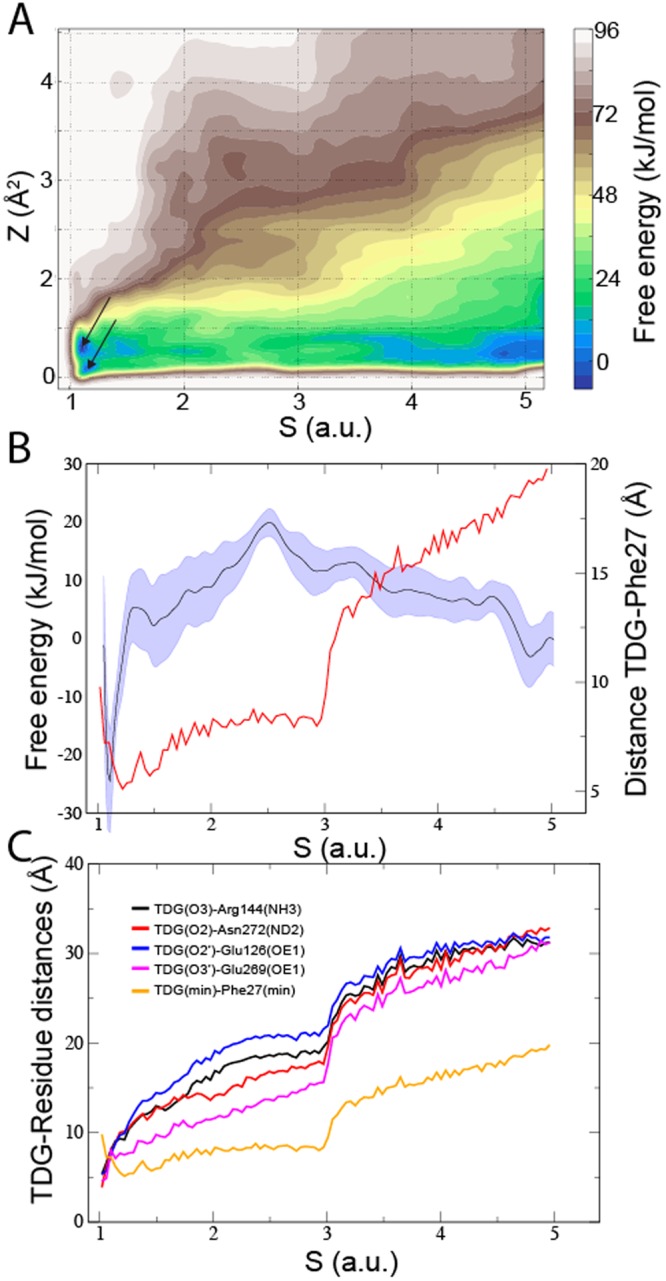
Free energy landscape associated with the periplasmic pathway. The free energies with respect to six reference positions of the TDG molecule in the simulated periplasmic pathway in (**A**) 2- and (**B**) 1-dimensions (blue). The arrows mark the two free energy minima. Standard errors correspond to variation in-between the free energy profiles resulting from the six parallel simulations. The average minimum TDG-Phe 27 distance is also shown (red). (**C**) The average minimum distances between TDG (oxygen atoms) and Phe 27 and binding residues Arg 144, Asn 272, Glu 126, and Glu 269 are shown with respect to the pathway (reference structures S = 1–6).

**Figure 6:**
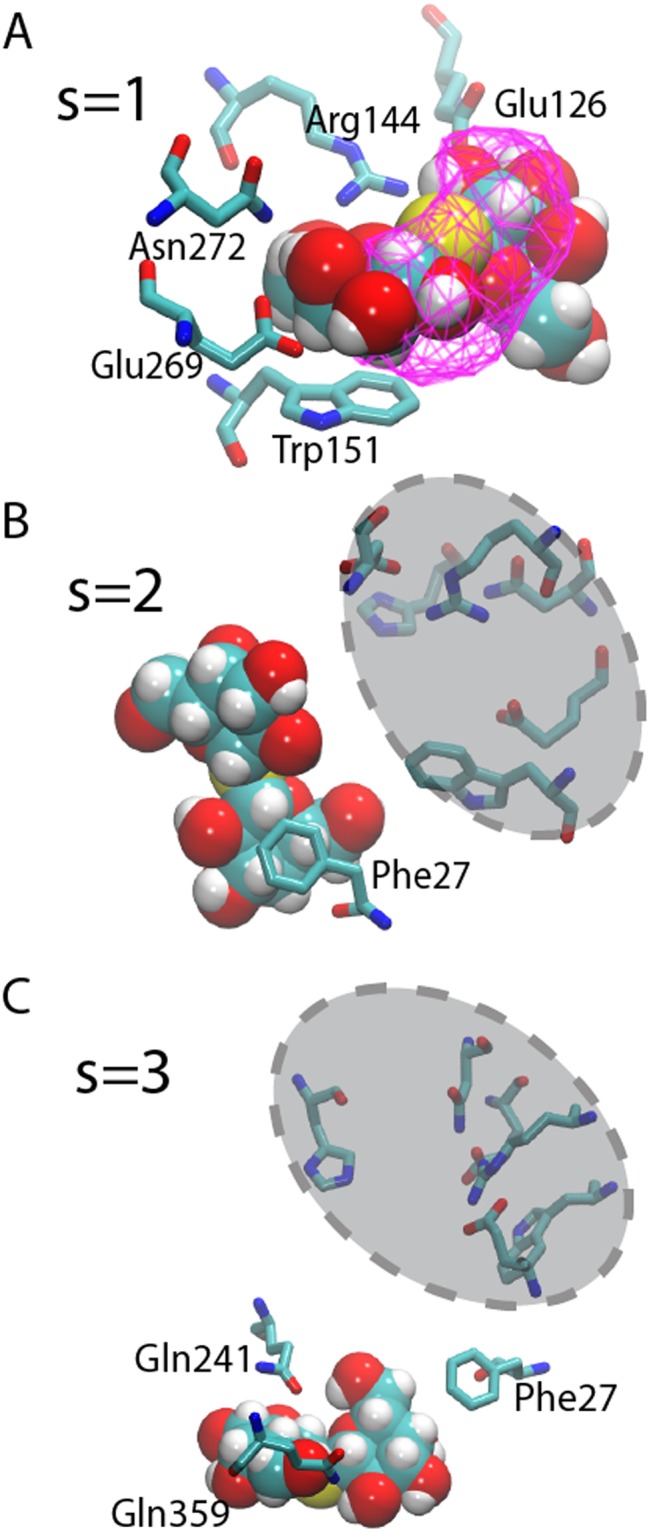
Representative metadynamics reference structures (**A**) An iso-density surface (magenta) of TDG extracted from 50 structures in the global free energy minimum is shown with respect to the binding site residues and TDG positions in the 4OAA crystal structure. (**B**) Reference point s = 2 is defined by ring-ring stacking between TDG and Phe 27 in the vicinity of the internal binding site (dashed shaded region). (**C**) Reference point s = 3 corresponds to TDG interacting with Gln 241 and Gln 359 before reaching Phe 27.

The free energy landscape showed favorable free energies at the periplasmic opening and the internal binding site, which were separated by a 20 ± 5 kJ/mol barrier (Fig. 5A,B) and an associated binding free energy of -24 ± 10 kJ/mol, which is in excellent agreement with the experimental binding free energy of −23 kJ/mol^24^. The free energy barrier was calculated as the difference between the global free energy minimum (reference point 1) and at S = 5.2, where TDG is located on the outskirts of the protein near bulk water (reference point 6). Two free energy minima (marked by arrows in Fig. 5A) were found in a location that corresponded to the binding position in the crystal structure, as can be observed by tracking distances between the TDG sugar and binding residues Arg144, Asn272, Glu126, and Glu269 (Fig. 5C). In addition, an iso-density surface calculated from 50 structures extracted from the global free energy minimum was aligned with the TDG binding pose in the crystal structure (Fig. 6A). To verify the free energy calculated using metadynamics, we also calculated the binding free energy of TDG using a thermodynamic cycle approach and arrived at −24 ± 4 kJ/mol, which is in agreement with both the metadynamics results (−24 ± 10 kJ/mol) and the experimental TDG binding free energy (−23 kJ/mol)^24^.

Because reference points 2 and 3 are located on each side of the free energy barrier, the corresponding structures hold clues to the molecular details that underlie the rise in free energy. Indeed, the protein-TDG interactions differ quite significantly in-between reference point 2 and 3 (Fig. 6). At reference point 3 (closer to the periplasm), the TDG was located at the periplasmic side of the narrowest section of the uptake pathway defined by Phe 27. Here, the sugar interacted with Gln 241 and Gln 359 in the wider periplasmic pore region and was in contact with the flexible side chain of residue Phe 27 (Fig. 6C). In reference point 2 (closer to the binding site), Phe 27 was stacked upon one of the TDG rings and the flexibility of Phe 27 enabled TDG to pass through the narrow opening separating the outer periplasmic vestibule from the binding site pocket (Fig. 6B). On average (over the six metadynamics simulations) sugar interactions with Phe 27 formed earlier than contacts to the binding residues (Fig. 5B,C). The preferred orientation of TDG also differs on each side of Phe 27. While the distribution of TDG orientation angles is evenly distributed at 0–180 degrees (TDG is symmetric) on the periplasmic side of Phe 27, the angle distribution shifts towards ~138 degrees inside of Phe 27 (Supplementary Fig. S3). This angle corresponds to TDG oriented along the membrane vertical to enable passage across the Phe 27 constriction. Finally, the free energy minima at the periplasmic end of the pathway can be attributed to favorable interactions to lipids in the periplasmic opening (Fig. 5A and Supplementary Fig. S4).

### Protein-substrate and lipid interactions

**Figure 7:**
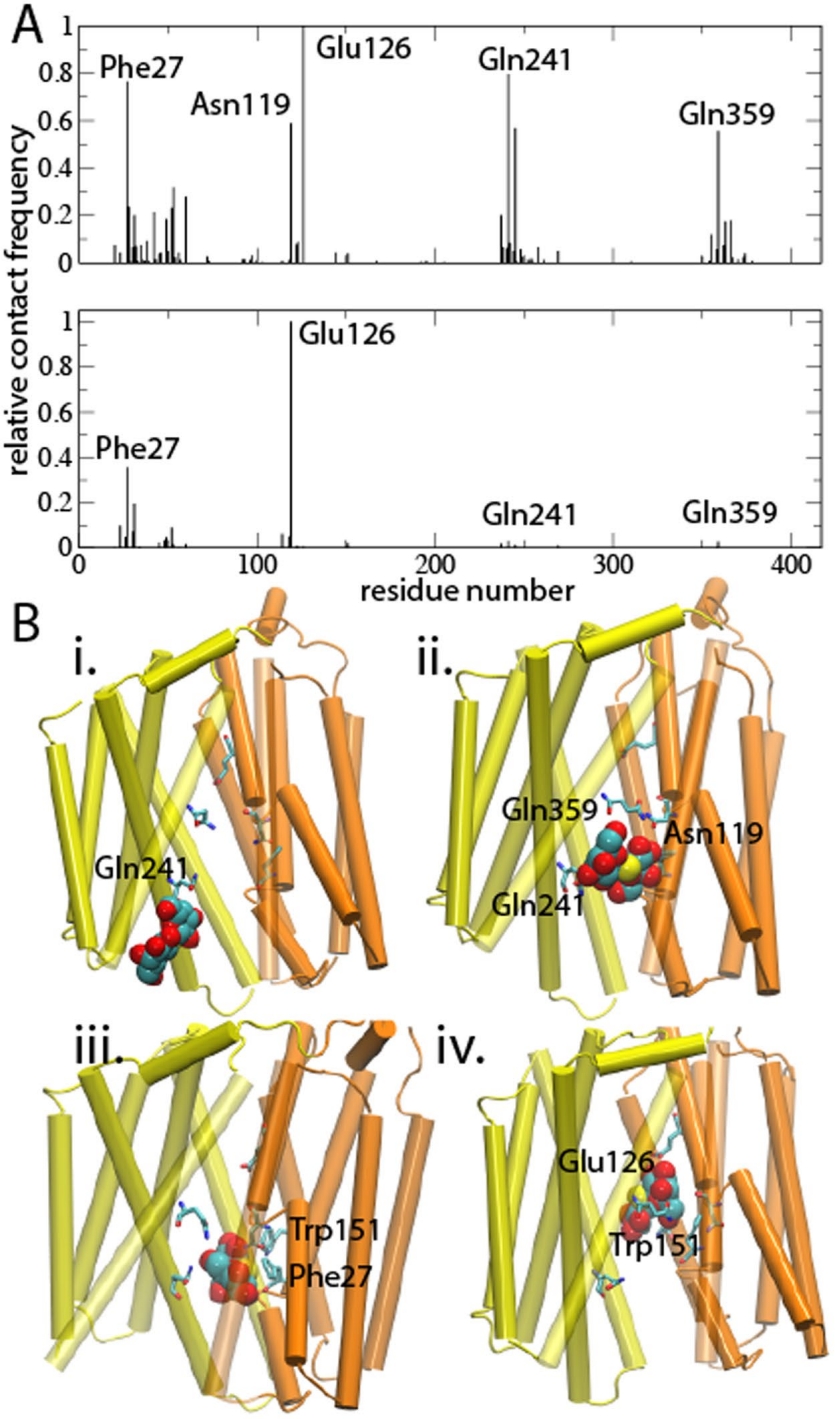
A putative uptake mechanism (**A**) Relative contact frequencies for interactions < 3.5 Å between TDG and protein in the TDG-bound (upper) and repeat (lower) simulations. (**B**) Representative simulation frames highlighting the TDG-protein interactions.

To extract the major relay nodes in the identified periplasmic pathway, we counted the number of interactions within 3.5 Å of TDG. In both the sugar-bound and repeat simulations, four high-contact regions, dominated by Phe 27, Glu 126, Gln 241, and Gln 359, were observed (Fig. 7A). Thus, even though no full exit was observed in the shorter repeat simulation, the TDG molecule was shuttled between identical relay nodes in the periplasmic pathway. We do not report a similar analysis for the multi-TDG simulation since here the TDGs spend most of the simulation time outside the protein, which would result in negligible contact scores.

Based on the observed results, we propose that lipids entering into LacY in-between helices 2 and 11 assist the initial stage in sugar uptake from the periplasm (Supplementary Fig. S4A,B). We do however not observe lipid perturbations in the vicinity of the protein leading to lipid deformations (Supplementary Fig. S4C) as observed for e.g. voltage-gated channels^27,28^. Subsequently, Gln 241 and Gln 359 attached to the substrate and guided it deeper into the periplasmic pathway (Fig. 7B, panel i-ii). We then observed a ring-ring stacking to Phe 27 (Fig. 7B, panel iii), which lines the narrowest portion of the periplasmic-open crystal structure^14^. Because the Phe 27 side chain is highly flexible, its reorientation enabled establishing a transient connection to Trp 151. The Phe 27 function is most likely unspecific shuttling via side-chain movements since the F27C mutant shows normal transport activity^16,17^. In our simulations, the Trp 151 interaction allowed release from Phe 27 and shuttling into the binding site region (Fig. 7B, panel iv) – dominated by Glu 126 in the contact analyses.

## Discussion

Proteins in the major facilitator superfamily (MFS) actively transport a range of different solutes across biological membranes using chemiosmotic gradients. While LacY has become the prototype system for studying secondary active transport, many questions remain unanswered. It is, for instance, not clear how the sugar substrate enters the transporter and what molecular rearrangements shuttle the sugar to the internal binding site. Recent crystal structures either display LacY in sugar-bound states, which seem occluded to the periplasm^12,13^ or in states where the periplasmic cavity is slightly more open^14,15^. These crystal structures displaying a more or less pronounced opening towards the periplasm were stabilized by either two double-Gly-to-Trp mutations or added nanobodies. Since periplasmic openings in all crystal structures were unexpectedly narrow and it was unclear whether the sugar substrate could be accommodated, one fundamental question that arises is what structural and chemical characteristics are required for the uptake channel to incorporate a sugar molecule and shuttle it towards the internal binding site.

The specificity of LacY stems from targeting the galactopyranosyl ring of lactose, which is the natural substrate. Because lactose affinity is relatively low, the sugar-bound crystal structures were determined in presence of high-affinity TDG and NPG substrate analogs^12,13,15^. The high-affinity analogs are incapable of triggering transport and were shown to, in combination with two double-Gly-to-Trp mutations, trap LacY in states that while occluded with respect to the sugar, showed slight openings towards the periplasm. Such states would, in principle, allow for hydration of the periplasmic pathway connecting the substrate-binding site with bulk water in the periplasm. Indeed, in our MD simulations of the TDG-bound crystal structure of LacY, the periplasmic pathway experienced immediate hydration. Extended sampling on the specialized Anton hardware enabled monitoring the bound TDG substrate exploring a putative uptake pathway and eventually exit into periplasmic bulk followed by re-entering. Spontaneous release and subsequent uptake enables non-biased characterization of the uptake channel. An early attempt to characterize such dynamics used biased sampling that resulted in obtrusive effects on the protein structure and, hence, the resulting protein-substrate interactions and free energy profile were questionable^23^. Despite significant sampling, we only observed one exit/re-enter event. However, upon repeating the simulation in the presence of multiple substrates in the bulk solvent, an identical uptake channel was observed.

The introduction of the wild-type sequence in the Gly-to-Trp double mutant TDG-bound structure did not result in significant changes at Gly 46, at the interface of helices H2 and H11 (Fig. 2A). However, the H5-H8 helix pair at Gly 262 underwent a ~7 Å movement to tightly seal the helix interface (Fig. 2B). Similar observations were made in a repeat simulation and for the simulated apo state (Fig. 2C). Because earlier simulations have identified protonation state-driven conformational changes in LacY^8,19^, the observed structural rearrangements could potentially be a consequence of protonation states in the sugar-binding site. The remaining H2-H11 opening towards the periplasm allowed residues Gln 241 and Gln 359 to direct sugar molecules towards Phe 27 and, subsequently, the internal binding site (Fig. 6). This explains early findings that identified helix 2 as part of a conformationally sensitive interface between the N- and C-terminal domains of the protein^29^.

The fucose transporter was the first MFS protein to be crystallized in an outward-facing conformation^30^. The structural rearrangements showed to achieve the outward-facing state were extensive. Therefore, the relatively subtle structural differences between the periplasmic-open LacY crystal structure and the sugar-bound structures were unexpected. Indeed, while the periplasmic cavity is capable of significant opening as shown by double electron-electron resonance (DEER)^31^, Förster resonance energy transfer (FRET)^32^, and cross-linking experiments^33^, our simulations suggest that sugar uptake is possible when the protein is slightly less open protein (Supplementary Fig. S5).

Furthermore, our simulations of substrate release and re-entering from the TDG-bound structure showed significant rearrangements from the kink region at Phe 27 toward the periplasm (Fig. 2D), which are in agreement with the observations from the crystal structures. Hence, the sugar-binding pocket appears confined by Phe 27, which also contributed the second largest number of interactions to TDG in the simulation (Fig. 7A) and a continuous rise in free energy associated with passage through the narrow pore (Fig. 5). Because the F27C mutant exhibits normal transport activity^16,17^, entering through the narrowest part of the uptake pathway is likely achieved by non-specific steric interactions. In addition, a sharp decline in TDG distances to Phe 27 preceded a gradual approach of the TDG towards the sugar-binding residues (Fig. 5), which indicate a shuttling role of Phe 27 enabled by the intrinsic side-chain flexibility.

Substrate shuttling by Phe 27 across the narrow passage separating the wide periplasmic pore and the internal binding site of LacY coincides with the free energy barrier. Hence, although TDG makes favorable protein interactions to e.g. Phe 27 at this passage, there is significant steric resistance. The magnitude of the free energy barrier is therefore likely related to the degree of opening at this narrow section, which is in agreement with observations that opening of the periplasmic cavity has been shown to be the rate-limiting step in the LacY reaction cycle^34^. Therefore, mutating Phe 27 to a significantly smaller side chain could possibly result in easier access to the binding site and an increase in transport rate.

Pore radii analyses of the sugar-bound occluded (PDB ID: 4OAA) and periplasmic-open (PDB ID: 5GXB) structures show both structures to display similar degrees of opening at Phe 27 (Fig. 2D). In contrast, several frames in the extended Anton simulation show ~2 Å increases in pore radii at the corresponding position, which explains why we observed spontaneous release and uptake of the TDG substrate. The high degree of flexibility observed in this region indicates that free energy barrier associated with passage by Phe 27 can undergo significant shifts. Hence, our simulations indicate that LacY has evolved a capability of displaying large structural fluctuations in particular from Phe 27 towards the periplasm to enable substrate uptake.

In all membrane protein transporters, the cargo needs to interact with water, protein, and lipids. For example, it has been shown that entropy changes induced by immobilized waters help drive conformational changes in LacY^35^. By tracking the interactions between TDG and water, protein, and lipids it was possible to discern a pattern associated with spontaneous release and uptake of the substrate. At instances in the simulation where TDG was located in the vicinity of the periplasmic opening, we observed bursts of lipid interactions (Fig. 3B). However, only at one of these instances did TDG exit the protein. Interestingly, bursts of TDG-lipid interactions correlated with temporary decrease in water hydration (Fig. 3AB). Hence, our computer simulations show that there is an intricate interplay between lipids, water and protein at the membrane interface that govern release and uptake of the substrate in LacY.

## Materials and Methods

### Building the systems

To construct the starting structure for simulation, the missing Ala 191-Ser 206 loop was modelled using the software Modeller^36^. The protonation states were assigned according to predictions based on the LacY apo protein structure by the software PROPKA3^37^ except for Glu 269, which was deprotonated in agreement with a 2.9 Å crystallographic oxygen-oxygen interatomic distance between Glu 269 and TDG (PDB ID: 4OAA)^13^. Instead, a proton was assigned to Glu 325 consistent with the proposed symport mechanism^3^. The protein was inserted into an equilibrated phosphatidylethanolamine (POPE) membrane^19^ and lipids were removed to avoid steric clashes resulting in a system consisting of 494 lipids, 18,485 water molecules, 59 Na^+^ and 66 Cl^−^ to achieve 0.15 mM concentration and an electrical neutral system. In the apo system, the TDG molecule was deleted before solvation. The system including multiple TDG molecules was built from the last frame of the 6.9 μs simulation by replacing water with 19 TDG molecules randomly distributed in the cytoplasmic and periplasmic bulk at least 10 Å from the protein.

### MD simulations

The LacY system was energy minimized and lipids and water molecules were relaxed as described earlier^19^. The protein was then equilibrated in a 50 ns simulation at constant temperature (310 K) and pressure (1 atm) (NPT ensemble). An identical system was simulated with a different random seed. The apo system was equilibrated similarly, while the multiple-TDG system was simulated for 1.3 μs. These MD simulations were run with the NAMD 2.9 software package^38^. The CHARMM22 including CMAP correction^39^ and CHARMM36^40^ force fields were used for protein and lipids, respectively, and the TIP3P model^41^ was used for the water molecules. The Multi-purpose Atom-Typer for CHARMM (MATCH)^42^ was used to obtain parameters for TDG. A time step of 1 fs was used to integrate the equations of motion, and a reversible multiple time step algorithm of 4 fs was used for the electrostatic forces and 2 fs for short-range, non-bonded forces. The smooth particle mesh Ewald method^43,44^ was used to calculate electrostatic interactions. The short-range interactions were cut off at 12 Å. All bond lengths involving hydrogen atoms were held fixed using the SHAKE^45^ and SETTLE^46^ algorithms. A Langevin dynamics scheme was used for thermostating and Nosé-Hoover-Langevin pistons were used for pressure control^47,48^.

The coordinates and velocities from the LacY and sugar-bound and systems were converted into the Anton-specified format and simulations were run on the Anton special-purpose supercomputer^25^ software version 2.4.1. The force fields were similar to the NAMD equilibration simulations. Pre-processor scripts guess_chem, refine_sigma, and subboxer optimized cut-off distances, electrostatic parameters, and spatial domain decomposition, respectively. The bonds between hydrogen and heavy atoms were constrained by the M-SHAKE algorithm^49^. Long-range electrostatic interactions were modelled by the Gaussian split Ewald (GSE) method^50^ with a 64 × 64 × 64 mesh. The RESPA multiple time-step method^51^ integrated the equations of motion every 6 fs and 2 fs for the electrostatic and all other interactions, respectively. The pressure was held at 1 atm using a semi-isotropic Berendsen barostat with a relaxation time of 2 ps and the temperature was maintained at 310 K with a Berendsen thermostat with τ = 1 ps.

The parameterization of TDG was further verified by calculating the absolute binding free energy of TDG to the receptor-binding site using a thermodynamic cycle, which consisted of two series of simulation. The solvation free energy, ΔG_solv_, was calculated with 20 simulations whereas TDG decoupling from the binding site, ΔG_complex_, was calculated with 30 simulations, each 1 ns long. These simulations span from TDG in full interaction with its environment to having van der Waals and Coulomb interactions turned off. The change in free energy of the system at each step-wise drop in interaction rate was calculated using the Bennett Acceptance Ratio implemented in GROMACS^52^. The binding free energy is determined as the difference in the total change in-between the two series of simulations. To ensure that TDG was positioned in the binding site, six restraints were imposed and later corrected for, ΔG_restr_, as described in Boresch *et al.*^53^. The calculations yielded ΔG_solv_ = 130.1 ± 1.2 kJ/mol, ΔG_complex_ = 175.0 ± 3.4 kJ/mol and ΔG_restr_ = 21.4 kJ/mol, which results in an absolute binding free energy of ΔG = ΔG_solv_ − ΔG_complex_ + ΔG_restr_ = −24 ± 4 kJ/mol.

### Metadynamics simulations

The metadynamics simulations used the PLUMED implementation^54^ and the Gromacs 5.1.2 engine^55^. The collective variables were based on RMSD to reference structures along the pathway observed in the Anton simulation, as defined in ref.^26^. As a result, the metadynamics simulations determine the free energy along the path, S, and along the divergence from the path, Z (Å^2^). Thus, each integer value of S marks a reference structure of TDG along the identified pathway, while the divergence, Z, can vary between zero Å^2^, for no divergence, to an unlimited value. To avoid bias towards the binding site in the crystal structure, reference S = 1 was chosen as the final frame from the 50 ns equilibration simulation. Reference S = 6 corresponded to TDG in bulk water and lipids with no protein interactions. References S = 2–5 were extracted in-between the end reference structures.

To restrict the explored conformational space and avoid artefacts from the periodic boundary conditions, boundaries were set at S = 5.2 and Z = 10 Å^2^, in the form of spring potentials with the spring constants set to 50 kJ/(mol∙Å) and 15 kJ/(mol∙Å), respectively. Because the spring potential was zero at S = 5.2, the corresponding free energy was used to calculate the barrier to the internal binding site. Repulsive Gaussian potentials with widths 0.01 (a.u.) along S and 0.5 (Å^2^) along Z with heights of 0.1 kJ/mol were deposited within the CV space at a 1 ps time interval. Six walkers (parallel simulations) were run from unique conformations evenly distributed along the path. The total simulation time for each walker was 235 ns, making a total of 1400 ns. Convergence can be deduced by the standard errors in-between the six simulation walkers (Fig. 5B).

### Data analyses

Data analyses were performed using VMD^56^. Pore radii were calculated with the software HOLE^57^ using 100 simulation frames extracted from the final 100 ns of the sugar-bound simulation along the membrane (z) axis and the end radius of the hard sphere probe was 5 Å.

## Electronic supplementary material

Supplementary Information

## Data Availability

The datasets generated during and/or analyzed in the current study are available from the corresponding author on reasonable request.
