# Supplementary material for: Uptake dynamics in the Lactose permease (LacY) membrane protein transporter
Source: Sci Rep. 2018 Sep 25;8:14324. doi: 10.1038/s41598-018-32624-7 (PMC6156506; doi:10.1038/s41598-018-32624-7)
Supplement: Supplementary file 1 — Supplementary Information [file 41598_2018_32624_MOESM1_ESM.docx]

SUPPLEMENTARY INFORMATION

**Uptake dynamics in the Lactose permease (LacY) membrane protein transporter**

D. Kimanius, Lindahl, E., Andersson, M.


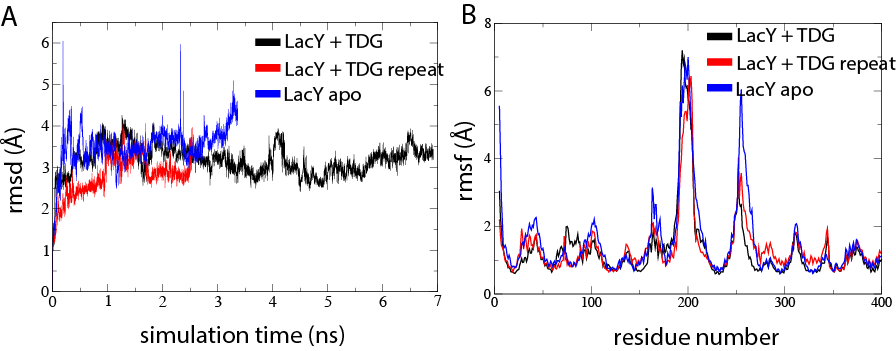


**Supplementary Figure S1.** Simulation dynamics. The root mean square deviation (rmsd) **(A)** root mean square fluctuation (rmsf) **(B)** for the LacY simulation with TDG (black), the repeat simulation of LacY with TDG (red), and the simulation of LacY with the co-crystallized TDG substrate removed (blue). The rmsf for the TDG system was comparatively low at position 262 (helix 8) because helix pair 5-8 closed early in the simulation and remained stable (low rmsf dynamics). The same helix pair did not close on this time scale in the TDG repeat simulation and hence generated an intermediate rmsf value. The helix pair closed in the apo simulation but over a significant sampling time, which generated a higher rmsf value.


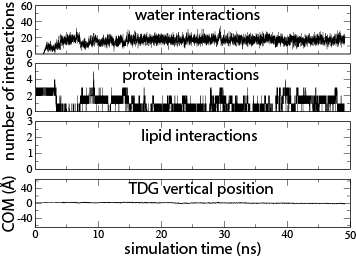


**Supplementary Figure S2.** Sugar interactions and in the equilibration simulation. The number of interactions to water, protein, and lipids within 3.5 Å of the TDG molecule are shown for the equilibration simulation. The lowest plot shows the center-of-mass position of the TDG molecule along the membrane vertical (z).


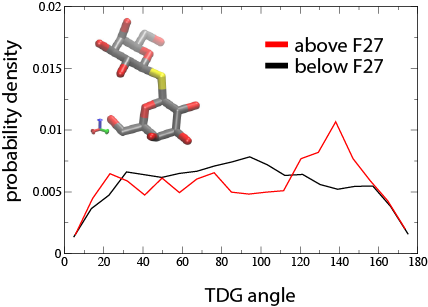


**Supplementary Figure S3.** Distributions of TDG angles with respect to the membrane vertical above (red) and below (black) Phe 27. The preferred ~138 degree orientation of TDG above Phe 27 is shown (inset).


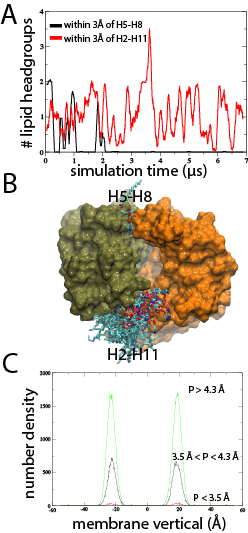


**Supplementary Figure S4.** Lipid interactions at the periplasmic entrance and membrane thickness **(A)** The number of lipid headgroup within 3 Å of helix pairs 5-8 and 2-11. **(B)** Cartoon showing specific lipids within 3 Å from helix pairs 5-8 and 2-11 in the sugar-bound simulation. LacY is viewed from the periplasm. **(C)** The number densities of lipid phosphates within 3.5 of the protein, in-between 3.5 Å and 4.3 Å, and outside 4.3 Å.


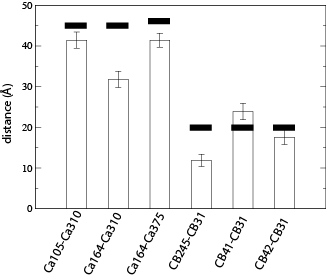


**Supplementary Figure S5.** Comparison to experimental mesurements of periplasmic opening. The black boxes correspond to the experimentally observed distances in all cases except the FRET distance between residues 164 and 375 of 60.4 Å, which is not displayed in the plot. Distances between residue pairs 105-310, 164-310, and 164-375 correspond to DEER experiments, while distances between pairs 245-31, 41-31, and 42-31 were obtained from cross-linking experiments. Simulation data were compared to the FRET and DEER experiments by comparing to distances between C-alpha (Ca) atoms of the corresponding pairs or in the case of cross-linking experiments to C-beta (CB) atoms after taking into account the CB-S linker offset (1.8 Å).

| Simulation # | Simulation time (μs) | Substrate |
| --- | --- | --- |
| 1 | 6.9 | TDG |
| 2 | 2.5 | TDG |
| 3 | 3.5 | - |
| 4 | 1.3 | TDG x 19 |
| 5-10 | 1.4 | TDG |

**Supplementary Table I**
